# Monoclonal Antibodies, Gene Silencing and Gene Editing (CRISPR) Therapies for the Treatment of Hyperlipidemia—The Future Is Here

**DOI:** 10.3390/pharmaceutics15020459

**Published:** 2023-01-30

**Authors:** Melody Hermel, Madison Lieberman, Leandro Slipczuk, Jamal S. Rana, Salim S. Virani

**Affiliations:** 1 United Medical Doctors, La Jolla, CA 92037, USA; 2 Department of Psychology, College of Arts & Sciences, Undergraduate, Georgetown University, Washington, DC 20057, USA; 3 Cardiology Division, Montefiore Medical Center, Albert Einstein College of Medicine, Bronx, NY 10461, USA; 4 Department of Cardiology, Kaiser Permanente, Northern California, Oakland, CA 94611, USA; 5 Health Policy, Quality & Informatics Program, Michael E. DeBakey VA Medical Center Health Services Research & Development Center, Houston, TX 77030, USA; 6 Section of Cardiology, Michael E. DeBakey Veterans Affairs Medical Center, Houston, TX 77030, USA

**Keywords:** atherosclerotic cardiovascular disease, cardiovascular prevention, hyperlipidemia, hypertriglyceridemia, lipoprotein (a)

## Abstract

Hyperlipidemia is a significant risk factor for atherosclerotic cardiovascular disease. Undertreatment of elevated lipids persists despite existing therapies. Here, we provide an update on monoclonal antibodies, gene silencing therapies, and gene editing techniques for the management of hyperlipidemia. The current era of cutting-edge pharmaceuticals targeting low density lipoprotein cholesterol, PCSK9, lipoprotein (a), angiopoietin-like 3, and apolipoprotein C3 are reviewed. We outline what is known, studies in progress, and futuristic goals. This review of available and upcoming biotechnological lipid therapies is presented for clinicians managing patients with familial hyperlipidemia, statin intolerance, hypertriglyceridemia, or elevated lipoprotein (a) levels.

## 1. Introduction

### An Area of Unmet Need

Hyperlipidemia remains of critical importance as a causal risk factor for atherosclerotic cardiovascular disease (ASCVD). Despite extensive research related to causes and treatments, hyperlipidemia remains underdiagnosed and undertreated [1,2]. Low density lipoprotein (LDL) is one of the main apolipoprotein B (Apo B) containing lipoproteins. Low density lipoprotein cholesterol (LDL-C), a component of the lipid profile, represents the total concentration of cholesterol within LDL, intermediate density lipoprotein (IDL) cholesterol and lipoprotein (a) particles, and has a particular importance for ASCVD, with the magnitude and duration of exposure increasing the risk [3]. Reducing LDL-C lowers cardiovascular (CV) risk, with estimates being a one fifth reduction in the annual rate of heart attack, revascularization, and ischemic stroke for each 1.0 mmol/L (approximately 39 mg/dL) reduction in LDL-C [4]. Moreover, LDL-C is one of the main risk factors to target for ASCVD disease prevention. LDL-C lowering therapies are widely available, yet the rates of hyperlipidemia are climbing. Indeed, global registry data have detected an exponential increase in the burden of elevated LDL-C over the past 25 years [5].

Notably, there are certain patients with particularly high-risk lipid profiles and even these most high-risk patient populations are diagnosed late and undertreated based on guideline recommended targets [6,7]. These high-risk groups include patients with severe hypercholesterolemia (LDL-C levels ≥ 190 mg/dL). For many of these patients, their LDL-C levels remain uncontrolled despite maximal doses of cholesterol lowering therapy, termed refractory hypercholesterolemia [8]. These classifications include familial hypercholesterolemia (FH), a condition impacting proteins in the LDL receptor pathways or other underlying genetic causes [9]. Another factor contributing to risk is sex, as women are underdiagnosed and undertreated as compared to men [10,11,12].

Current guidelines suggest starting statin therapy as a first line agent for patients who meet treatment criteria for hyperlipidemia. For example, according to the American College of Cardiology/American Heart Association (ACC/AHA) guidelines, patients with clinical evidence of ASCVD, severe hypercholesterolemia (LDL-C ≥ 190 mg/dL), patients aged 40–75 years with diabetes, elevated ASCVD risk based on a 10-year risk calculation, or other risk-modifying factors should be started on statin therapy following a risk discussion [13]. Similarly, guidelines from the European Society of Cardiology (ESC) and European Atherosclerosis Society (EAS) advise treatment with statins as a first line agent [14]. However, despite these guidelines and the wide availability of effective statin therapy, many patients still have severe hypercholesterolemia (LDL-C levels ≥ 190 mg/dL), sometimes refractory to maximal medical therapy. In part, this is due to adverse effects limiting patients’ ability to tolerate the recommended intensity of statin therapy, poor compliance, and poor response to treatment related to individual genetic differences, or lack of recognition/aggressive treatment in women and ethnic minorities [15,16,17,18,19,20]. Furthermore, patients with the above-mentioned high-risk conditions may have extremely high LDL-C making it very difficult to reach aggressive targets set out by some guidelines. Non-statin agents may be used to augment statin therapy. However, this combination therapy is often nevertheless insufficient [21,22,23,24,25,26].

Clinicians now have broader treatment options beyond statin therapy and traditional non-statin agents. Recent advancements in lipid lowering therapies include monoclonal antibodies, gene silencing therapy, and gene editing therapy. Importantly, these non-statin options target both LDL-C and non-LDL-C pathways which also play a role in ASCVD. Indeed, lipoprotein (a) (Lp (a)) and hypertriglyceridemia have been recognized as independent risk factors for ASCVD [24,27,28,29,30,31,32]. These therapies have also moved genetics from being a traditionally nonmodifiable ASCVD risk factor to being a feasible drug therapy target in the imaginable future. A review of these three major domains of cholesterol therapies will equip the reader with an understanding of opportunities to optimize patient care in this area of greatly unmet need

## 2. Monoclonal Antibodies

**Figure 1:**
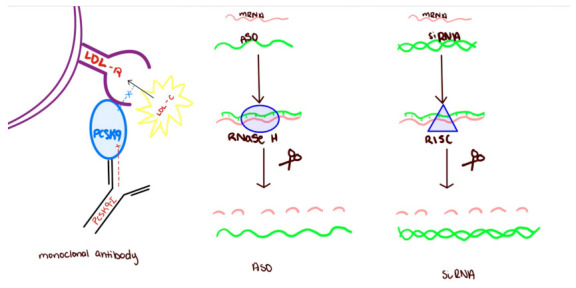
Mechanism of action of monoclonal antibodies and gene silencing techniques.

Modeled after immunoglobulin G (IgG) and humoral immunity, monoclonal antibodies are IgG based molecules designed to attach to a specific target (see Figure 1). Initially applied in cancer medicine, the first monoclonal antibodies were hybrid between murine myeloma cells and splenic B lymphocytes [33]. The murine origin of the monoclonal antibodies made them non-sustainable due to the development of human anti-murine antibodies. However further development of monoclonal antibodies led to the development of chimeric clones with human crystallizable fragments as opposed to murine ones. Next, the process of “humanization” allowed murine protein loops to be implanted with human immunoglobulins. Modern day monoclonal antibodies are fully human which minimizes adverse effects.

### 2.1. Target: Proprotein Convertase Subtilisin/Kexin Type 9 (PCSK-9)

The debut of monoclonal antibodies in dyslipidemia management began with the advent of the PCSK-9 inhibitors. In 2003, the significance of the PCSK9 protein in lipid metabolism was discovered [34]. The PCSK9 protein interacts with LDL receptors on the hepatocyte surface and prevents the receptors resurfacing and recycling. LDL particles are normally eliminated from the blood stream by binding to LDL receptors on the hepatocyte surface. By promoting intracellular degradation of the LDL receptor, the PCSK9 protein allows more LDL particles to circulate in the bloodstream. Disturbing this critical modulation of receptor levels, PCSK9 inhibitors prolong the life span of the LDL receptor and reduce plasma LDL-C [35]. Very low LDL-C values have been found in patients with a loss of function mutation of PCSK9 protein, while gain of function mutation of the PCSK9 protein is associated with higher plasma LDL-C levels [36,37,38]. Importantly, PCSK9 inhibitors have been noted to reduce LDL-C without altering inflammatory markers, such as high-sensitivity C-reactive protein (hs-CRP) [39]. Still, studies have shown that elevated hs-CRP may identify patients at higher risk who would benefit the most from PCSK9 inhibitors [40,41,42].

In the OSLER and ODYSSEY LONG TERM trials, the PCSK9 inhibitors alirocumab and evolocumab used on top of moderate or high intensity statins reduced LDL-C levels by an additional 60% and were well tolerated [43,44]. Following this, the FOURIER trial was conducted in a prospective randomized fashion to determine if evolocumab would lead to a reduction in CV events in patients with stable ASCVD [45]. The FOURIER trial randomized 27,564 patients with ASCVD and LDL-C > 70 mg/dL or non-HDL-C of ≥100 mg/dL on optimized dose of a statin to evolocumab (13,784 patients) or placebo (13,780 patients) either every two or four weeks. At 2.2 years of follow-up, the primary end point (the composite of CV death, myocardial infarction (MI), stroke, hospitalization for unstable angina, or coronary revascularization) was significantly reduced in the evolocumab group (1344 patients (9.8%)) as compared to placebo (1563 patients (11.3%)); hazard ratio, 0.85; 95% confidence interval (CI), 0.79 to 0.92; *p* < 0.001. Aside from mild injection site reactions, there was no significant difference in side effects between the two groups. In the recently published FOURIER-OLE trial, 6,635 patients were treated with evolocumab every two or four weeks regardless of their treatment group in the parent trial [46]. Patients who received evolocumab in the parent trial and continued its use in the FOURIER-OLE trial had 23% lower risk of CV death as compared to patients who took placebo in the parent trial (HR 0.77, 95% CI 0.60–0.99, *p* = 0.04), and there was a 15% reduction in the primary endpoint from the parent trail (HR 0.85, 95% CI 0.75–0.96, *p* = 0.008). Benefit from PCSK9 therapy persisted over time. Furthermore, the ODYSSEY OUTCOMES trial demonstrated the safety and efficacy of alirocumab as compared to placebo for patients with an ACS event in the preceding year on maximally tolerated statin therapy. After an initial run-in phase of 2–16 weeks on high-intensity statin therapy, a total of 18,924 patients were enrolled and received either alirocumab 75–150 mg every two weeks (to maintain LDL-C between 25 and 50 mg/dL) or placebo. In this trial there was a significant reductionmajor adverse cardiovascular events (MACE) for subjects treated with alirocumab vs. placebo (9.5% vs. 11.1%, HR 0.85, 95% CI 0.78–0.93, *p* < 0.001).

Alirocumab has also been associated with greater reduction in plaque burden and plaque regression for secondary prevention in high-risk patients. In the PAC-MAN-AMI trial, 300 patients with history of MI status post urgent PCI on high intensity statin therapy were randomized to alirocumab 150 mg or placebo every two weeks for a duration of 52 weeks. Within 24 h of PCI, patients received the first injection. Intravascular ultrasonography (IVUS), near-infrared spectroscopy, and optical coherence tomography were performed in the remaining arteries without the culprit lesion at baseline and after 52 weeks. Mean change in percent atheroma volume was −2.13% with alirocumab vs. −0.92% with placebo (difference, −1.21% (95% CI, −1.78% to −0.65%), *p* < 0.001). Patients with percent atheroma volume regression were 84.6% among those receiving rosuvastatin 20 mg plus alirocumab vs. 65.9% among those receiving rosuvastatin 20 mg plus placebo, *p* < 0.001. Adverse events were equivalent in the two groups. Moreover, the GLAGOV and HUYGENS trials also found favorable plaque changes with evolocumab [47,48]. The GLAGOV trial was a randomized double-blind, placebo-controlled study that enrolled 968 patients with symptomatic CAD on statins or combination therapy with evolocumab and statins. Results noted a mean change in percent of atheroma volume of about 1% (*p* < 0.001), with regression induced in an even greater percentage of patients. The HUYGENS trial, patients with a non–ST-segment elevation MI were treated with monthly evolocumab 420 mg (n = 80) or placebo (n = 81) for 52 weeks and underwent serial OCT and intravascular ultrasound imaging within a matched arterial segment of a nonculprit vessel. Results showed a greater regression of percent atheroma volume in the evolocumab versus placebo groups (−2.29% ± 0.47% vs. −0.61% ± 0.46%; *p* = 0.009).

Alirocumab and evolocumab have been approved by the Food and Drug Administration (FDA) as an add on to lifestyle modification and statin therapy for the treatment of individuals with primary hyperlipidemia including adult patients with heterozygous or homozygous FH or for secondary prevention in patients with existing ASCVD [49,50]. Evolocumab is also approved for homozygous FH in children > 10 years of age. In the 2018 AHA/ACC/Multi-society cholesterol guidelines, PCSK9 inhibitors are considered for primary prevention in patients with heterozygous FH and elevated LDL-C (>100 mg/dL) despite high intensity statin therapy and ezetimibe. Furthermore, in patients with hypercholesterolemia (baseline LDL-C > 220 mg/dL), between ages 40 and 75 with LDL-C above 130 mg/dL, despite maximally tolerated statin and ezetimibe therapy, PCSK9 inhibitors may also be utilized. In addition to lifestyle modification and statin therapy, PCSK9 inhibitors are considered for secondary prevention in patients at very high risk for ASCVD events whose LDL-C remains ≥ 70 mg/dL or whose non-HDL-C level remains ≥100 mg/dL despite maximally tolerated lipid-lowering agents (statins and ezetimibe) [13]. Very high-risk is defined as having a history of multiple ASCVD events (recent ACS within the past 12 months, history of MI outside of the ACS event, history of ischemic stroke, or symptomatic PAD) or one major ASCVD event plus multiple high-risk conditions (age ≥ 65 years, heterozygous FH, history of coronary revascularization outside of the major ASCVD events, diabetes mellitus, hypertension, chronic kidney disease, current smoking, persistently elevated LDL-C ≥ 100 mg/dL despite maximally tolerated statin and ezetimibe, or history of congestive heart failure).

Of note, there was a third humanized PCSK9 inhibitor, bococizumab, which was studied in the SPIRE program trials. The SPIRE program consisted of six parallel, multinational lipid-lowering studies and the SPIRE-1 and SPIRE-2 outcome trials based on CV events. Unlike the fully human monoclonal antibodies alirocumab and evolocumab, bococizumab contained small segments of murine sequence. In the SPIRE lipid-lowering studies, bococizumab was injected subcutaneously at a dose of 150 mg every two weeks and reduced LDL-C at 12 weeks by 55.2 percentage points compared to placebo. Of note, this reduction in LDL-C was significantly attenuated over time due to the development of high-titer anti-bococizumab antibodies. At one year, 48% of the patients who received bococizumab demonstrated detectable antidrug antibodies. In fact, LDL-C was only reduced 12% from baseline at 52 weeks among those individuals with the highest antibody titers. Moreover, there were higher rates of injection-site reactions (12.7 per 100 person-years) when compared to rates previously reported with evolocumab or alirocumab. Of significant concern was the wide variability in the LDL-C lowering effect from bococizumab which was present even before the detection of antidrug antibodies in patients [51].

In SPIRE-1, after seven months of follow-up, no significant difference was detected in the primary endpoint (non-fatal MI, non-fatal stroke, hospitalization for unstable angina requiring urgent revascularization, or CV death)among patients with LDL-C ≥ 70 mg/dL (1.8 mmol/L) randomized to 150 mg bococizumab every two weeks vs. placebo (HR 0.99, 95% CI 0.80–1.22; *p* = 0.094). SPIRE-2 enrolled patients with LDL-C of ≥100 mg/dL (2.6 mmol/L) and demonstrated a significant risk reduction in the primary endpoint (HR 0.79; 95% CI 0.65–0.97; *p* = 0.02), suggesting that treating a higher risk cohort over a longer period may result in a clinical benefit. As noted above, the drug development was stopped early due to the development of antidrug antibodies in a large number of patients limiting the lipid lowering impact of the drug [51,52].

Factors that limit that use of PCSK9 inhibitors include cost, insurance authorization, and the requirement for frequent injections [53,54]. Currently, an oral PCSK9 inhibitor, MK-0616, is undergoing evaluation. At the 2022 AHA scientific sessions, data were presented from two small phase I randomized double-blind placebo-controlled trials assessing the optimal dose, safety, and pharmacokinetics of MK-0616 used along with permeation enhancers to facilitate absorption. In the first trial, 60 healthy male adult subjects were randomized to once daily oral MK-0616 of varying doses or placebo for eight weeks duration. There were no serious adverse events detected and mild side effects included gastrointestinal symptoms, headache, and rash. Free plasma PCSK9 was reduced by a maximum of 90%. In the second trial, MK-0616 was given to patients who were taking moderate to high intensity statins for at least three months. The trial included 40 patients randomized to receive once-daily dose of 10 or 20 mg of MK-0616 along with varying permeation enhancers or placebo and followed for 14 days. There were no serious side effects and LDL-C levels were reduced by about 65% after 14 days of treatment in patients who received MK-0616 [55,56]. Real-world efficacy and CV outcomes trials remain to be performed. However, these initial results may allow PCSK9 inhibitors to overcome treatment and adherence barriers [55,57].

Questions surrounding adverse cognitive effects of very low LDL-C < 20 mg/dL have surfaced despite the lack of evidence of such an association. The EBBINHAUS study analyzed a subgroup of patients from the FOURIER trial. Patients with stable ASCVD were randomized to receive either evolocumab or placebo as well as statin therapy and cognitive test scores were compared. Over a median of 19 months, there was no significant difference in cognitive function test scores or in subjective self-assessments of everyday cognition. Executive function, working memory, episodic memory, and psychomotor speed were not significantly different between groups [58,59]. Neither evolocumab nor LDL-C level was associated with impaired cognitive function. It is important to note that this study only assessed cognitive function at a single time point and longer-term data beyond 19 months are needed.

### 2.2. Target: Angiopoietin-Like Protein 3 (ANGPTL3)

Monoclonal antibodies have further been applied in the management of hypercholesterolemia via inhibition of ANGPTL3. ANGPTL3 is a protein which inhibits lipoprotein lipase and endothelial lipase, enzymes that degrade lipoproteins. People with loss of function mutations in the ANGPTL3 gene have been noted to have low plasma triglycerides, LDL-C, high-density lipoprotein cholesterol (HDL-C) and 41% lower risk of CAD than the general population [60,61,62,63]. By inhibiting lipoprotein degradation, ANGPTL3 regulates plasma lipid levels [64]. As this mechanism is independent of the LDL receptor, the ANGPTL3 pathway has been especially useful in patients with homozygous FH with no functioning LDL receptors, where statins and PCSK9 inhibitor may not be very effective therapies to lower LDL-C.

Evinacumab is an ANGPTL3 inhibitor that has been approved by the FDA for the treatment of adult and pediatric patients aged 12 years and older with homozygous FH, refractory hypercholesterolemia, and severe hypertriglyceridemia [65]. An initial phase I single ascending dose trial involving 83 subjects with TG (150–450 mg/dL) or LDL-C (≥100 mg/dL), who were randomized 3:1 to a single dose of evinacumab or placebo demonstrated dose dependent reductions in triglycerides (TG), non-HDL-C, LDL-C, and HDL-C with evinacumab [66]. The greatest reductions in TG were noted after administration of 10 mg/kg IV evinacumab with 88% reduction in TG compared to placebo. Adverse events did not significantly differ in treatment vs. placebo groups. Additionally, a phase I multiple ascending dose study of 56 subjects with TG (150–500 mg/dL) or LDL-C ≥ 100 mg/dL randomized 3:1 to evinacumab at varying doses subcutaneously once weekly, every two weeks, or intravenously once every four weeks up to day 56 revealed a median reduction in TG and VLDL-C of ~70% at day 57 in the 300 mg SC every week, 450 mg SC every week, and 20 mg/kg IV every four week groups [66]. LDL-C was also significantly reduced in all dosing groups, most notably in the 300 mg SC every week (22.0%, *p* = 0.0194) and the 20 mg/kg IV groups (25.1%, *p* = 0.0074). No serious adverse events were noted.

Following this, a phase 2, single group, open-label study was conducted in which nine adult patients with homozygous FH (including two null homozygotes and one compound heterozygote with two null alleles) were treated with evinacumab 250 mg subcutaneous on day 1 and then 15 m/kg intravenous on day 15 on top of their existing aggressive lipid-lowering medications. There was a 49 ± 23% (range, 25 to 90%) mean reduction in LDL-C from baseline to four weeks of follow-up, with an absolute decrease from baseline of 157 ± 90 mg/dL (range, 71 to 323 mg/dL) [67]. Apolipoprotein B, non-HDL cholesterol, TG, and HDL-C also decreased during the four weeks of follow-up. There were no serious adverse events. Another phase 2, double-blind placebo-controlled trial randomized 272 patients to subcutaneous or IV formulations of evinacumab of various doses and analyzed percent change in LDL-C from baseline. This study included patients with or without FH and refractory hypercholesterolemia with a screening LDL-C level of 70 mg/dL or greater with history of atherosclerosis or of 100 mg/dL or greater without evidence of atherosclerosis. Both formulations of evinacumab demonstrated LDL-C reductions greater than placebo in a dose dependent manner after 16 weeks of follow-up [68]. Thereafter, a phase 3 double blind placebo-controlled trial was designed to assess the percent change in LDL-C from baseline to 24 weeks of follow-up [69]. Sixty-five patients with homozygous FH on existing lipid lowering therapy were randomized in a 2:1 ratio to receive an intravenous infusion of evinacumab (15 mg/kg of body weight) every 4 weeks or placebo. Treatment with evinacumab resulted in a 49% reduction (CI from –65% to −33%; *p* < 0.001) in LDL-C compared to placebo, with the absolute difference in LDL-C of –132 mg/dL (95% CI, –175.3 to –88.9; *p* < 0.001) in treatment versus placebo groups. Rates of adverse events were equivalent between groups.

Menglong et al. conducted a meta-analysis of five randomized control trials, with healthy volunteers included in three of the trials and patients with hypercholesterolemia in two of the trials. They found that evinacumab was able to reduce LDL-C significantly compared with placebo (MD −33.12%, 95% CI −48.63% to −17.60%, *p* < 0.0001), triglycerides (MD −50.95%, 95% CI, −56.55% to −45.36%, *p* < 0.0001), and HDL-C (MD −12.77%, 95% CI, −16.35% to −9.18%, *p* < 0.0001) with no significant differences in serious adverse events between the placebo and treatment groups (see Table 1) [70].

## 3. Gene Silencing: Antisense Oligonucleotides (ASO)

Antisense Oligonucleotides (ASO) gained recognition in 1978 when they demonstrated the ability to block Rous sarcoma virus 35 translation. Hundreds of ASO based clinical trials have ensued since then with improvements in the design over time. The first iteration of ASOs were long single strand nucleotides complementary to an mRNA target which activated RNase H leading to mRNA degradation (see Figure 1). Newer ASOs have multiple different strategies to modulate protein translation involving chemically modifying different aspects of the RNA structure from the nucleotides to the sugars to the atom linkages and backbones that do not involve activation of RNase H [71,72,73]. Drug delivery to the liver is facilitated by a conjugate such as N-acetylgalatosamine carbohydrates (GalNAc). The use of GalNAc reduces the amount of drug necessary by targeting it to the liver and also reduces the chances of off-target effects.

### 3.1. Target: PCSK9

AZD8233 is an ASO targeting PCSK9 which is currently undergoing phase 2 clinical trials to test efficacy and safety. The initial phase 1 study of this agent administered ION449 (AZD8233) subcutaneously in multiple ascending doses in patients with dyslipidemia with or without type 2 diabetes. The ETESIAN phase 2b parallel, double-blind, placebo-controlled, dose-ranging study randomized 119 patients ages 18–75 with LDL-C of ≥70 and <190 mg/dL and fasting triglycerides of <400 mg/dL on statin therapy in a 1:1:1:1 fashion to subcutaneous injections (days 1, 8, 29, and 57) of AZD8233 90 mg, 50 mg, 15 mg or placebo. Patients were followed for up to 16 weeks after the last dose. LDL-C and PCSK9 levels were reduced in a dose dependent manner at week 12 for patients on study drug vs. placebo. LDL-C decreased −72% (95% CI −78 to −65) for the 50 mg dose and −79% (95% CI −83 to −74) for the 90 mg dose. There were no significant adverse events. The SOLANO study was a randomized double-blind placebo-controlled phase 2b trial in subjects with hypercholesterolemia who received 60 mg of AZD8233 every month and achieved 62.3% decrease in LDL-C compared to placebo after 28 weeks. The study demonstrated the safety and tolerability of the drug. An oral ASO is currently under investigation and has demonstrated effective plasma PCSK9 and LDL-C lowering in cynomolgus monkeys [74].

### 3.2. Target: ANGPTL3

Another approach to ANGPTL3 inhibition involves ASO therapy. In a phase I trial, an ASO targeting ANGPTL3 mRNA was assessed for its impact on triglyceride clearance, liver triglyceride content, insulin sensitivity, and atherosclerosis in mice. Then, 44 human subjects with mild to moderately elevated triglyceride levels were randomized to subcutaneous injections of placebo or an ASO targeting ANGPTL3 mRNA in varying single dose or multiple dose regimens. Safety, pharmacokinetics and pharmacodynamics, and lipid and lipoprotein levels were assessed [75]. After six weeks of follow-up, patients in the multiple dose groups had triglycerides reduced up to 63.1% and LDL-C reduced up to 32.9%. No serious adverse events were reported. A double-blind placebo-controlled phase 2 trial examined a GalNAc-conjugated ASO targeting ANGPTL3 termed vupanorsen. In this trial, 105 patients were included who had hypertriglyceridemia (>150 mg/dL), type 2 diabetes, and hepatic steatosis and randomized to subcutaneous vupanorsen or placebo in varying doses to assess percent change in triglycerides from baseline. With vupanorsen 80 mg monthly, patients had up to a –53% (95% CI –43 to –60% *p* value < 0.0001) decrease in triglycerides compared to placebo after six months. There was no reduction in LDL-C [76].

In the TRANSLATE-TIMI 70 double-blind placebo-controlled phase 2b study, vupanorsen was tested in adults on statins with non-HDL-C ≥ 100 mg/dL and triglycerides 150 to 500 mg/dL [77]. Two hundred eighty-six participants were randomized to varying vupanorsen doses vs. placebo biweekly or monthly. Vupanorsen achieved a decrease in non-HDL-C of up to 27.7% in a group receiving 80 mg every two weeks (*p* < 0.001). Triglycerides were reduced up to 56.8% (*p* < 0.001) in a dose dependent manner and LDL-C was reduced up to 16% without a dose dependent association.

### 3.3. Target: Lp (a)

Elevated Lp (a) is a genetically determined independent risk factor for early onset ASCVD and calcific aortic stenosis [71,72]. This particle accounts for substantial residual CVD risk beyond standard lipid levels and has been shown to be generally resistant to lifestyle changes and pharmacologic therapies like statins [32,78]. Lp (a), which consists of apoB covalently bound to apo (a) via a disulfide bond is extremely atherogenic and promotes inflammation. Some therapies have moderate Lp (a) lowering effects (niacin and PCSK9 inhibitors). Lipoprotein apheresis can also lower Lp (a), but this therapy is cumbersome and costly [30,31]. There are currently no FDA approved pharmacologic strategies to manage elevated Lp (a) concentrations. There are multiple pharmacological RNA based gene silencing agents being developed to address this need.

Pelacarsen is an antisense oligonucleotide which blocks Lp (a) production in the liver. In a phase IIb trial, 286 subjects were randomly assigned to groups of cumulative monthly doses of 20–80 mg pelacarsen versus placebo. Significant reductions in direct Lp (a) cholesterol in a dose-dependent manner were observed with pelacarsen compared with pooled placebo, by a mean of 29–67% versus 2% respectively; *p* = 0.001. Pelacarsen was also associated with a modest decrease in laboratory-reported LDL-C [79].

The Lp (a) HORIZON trial is a phase 3 trial currently ongoing. This global, multicenter, double-blind, placebo-controlled study is planned to assess patients with existing CVD treated with statin therapy and elevated Lp (a) levels > 60 mg/dL (150 nm/L) at the screening visit. Pelacarsen is given subcutaneously on a monthly basis at a dose of 80 mg. The study will assess whether the use of pelacarsen is associated with a reduction in the risk of MACE, including CV death, non-fatal MI, no-fatal stroke, and urgent coronary revascularization requiring hospitalization, with results expected in 2025 [80].

### 3.4. Target: APOC3

Triglyceride-rich lipoproteins and their remnants have been identified as important contributors to ASCVD [81]. Apolipoprotein C-III (apo C-III) is a glycoprotein found on VLDL, particles. Apo C-III inhibits the breakdown of triglyceride-rich lipoproteins by lipoprotein lipase [82,83]. In studies of patients with loss of function mutations of APOC3, mutation carriers had lower levels of TR, higher HDL-C, lower, LDL-C, and lower risk for coronary heart disease compared to non-carriers [84,85].

The ASO targeting APOC3, volanesorsen (previously called ISIS 304801, ISIS-ApoCIIIRx and IONIS-ApoCIIIRx), reduced apoC-III and TG levels [86]. Pooled analysis of four studies showed significant reduction in TG after three months of treatment with volanesorsen compared with placebo (MD: −73.9%; 95% CI: −93.5%, −54.2; *p* < 0.001 I2 = 89.05%; *p* < 0.001) with significant decrease in LDL-C and increase in HDL-C as well [87]. Still, there were safety concerns related to thrombocytopenia and bleeding. The US Food and Drug Administration (FDA) withheld drug approval for the treatment of patients with familial chylomicronemia based upon these safety concerns [88]. In 2019, volanesorsen was approved in the EU for the treatment of adult patients with familial chylomicronemia syndrome based on positive results from the APPROACH and COMPASS trials (see Table 2) [89,90].

### 3.5. Target: ApoB-100

Mutations in the apolipoprotein B (apoB-100) gene are associated with FH. Mipomersen is an ASO targeting apoB-100. During phase I, II, and III trials, mipomersen significantly lowered LDL-C, apoB-100, and Lp (a) from baseline with main side effects consisting of injection site reactions, flu-like symptoms, and hepatotoxicity. In a phase 3, multicenter, blinded, randomized, placebo-controlled study, mipomersen reduced LDL-C by −36.9% as compared to the placebo group at −4.5% (*p* < 0.001). Specifically, this trial included 158 patients with LDL-C ≥ 100 mg/dL and a history of or high risk for coronary heart disease at trial enrollment. Patients received 200 mg mipomersen or placebo on a weekly basis subcutaneously for 26 weeks and then were followed for 24 weeks [91].

## 4. Gene Silencing: Short Interfering RNA (SiRNA)

Unlike ASOs, short interfering RNA (siRNA) are double-stranded RNA sequences containing a guide strand and a passenger strand (see Figure 1). An siRNA targeting a specific protein is subcutaneously administered, attached to a sugar, GalNaC (an aid in transport into the liver). The guide strand binds to RNA-induced silencing complex (RISC) in the cytoplasm. The RISC complex uses the guide strand to find and cleave the complementary mRNA sequence [92,93].

### 4.1. Target: PCSK9

Inclisiran is the first in class siRNA to receive FDA approval as an add on to lifestyle modification and statin therapy to treat patients with existing ASCVD [94]. In blocking the translation of the PCSK9 protein in the liver, inclisiran lowers plasma LDL-C levels. In the phase 3 double blind randomized ORION-9 trial, 482 adults with heterozygous FH (with LDL-C level > 100 mg/dL at time of enrollment) were assigned in a 1:1 ratio to receive subcutaneous inclisiran sodium 300 mg or placebo at specific time points. After 18 months, LDL-C was reduced by −39.7% (95% CI −43.7 to −35.7%) for those who received inclisiran versus a small increase in LDL-C in the placebo group with an absolute difference of −47.9 percentage points between the groups (95% CI −53.5 to −42.3%; *p* < 0.001). Side effects were equivalent in the two groups [95]. The ORION-10 and ORION-11 trials were randomized, double-blind, placebo-controlled, parallel-group, phase 3 trials which studied the efficacy and safety of inclisiran in high-risk participants with a history of ASCVD and refractory hypercholesterolemia despite maximally tolerated statin therapy. ORION-10 enrolled adults in the US with ASCVD, while ORION-11 enrolled adults in Europe and South Africa with ASCVD or an ASCVD risk equivalent (type 2 diabetes, FH, or a 10-year risk of a CV event of ≥20% as assessed by the Framingham Risk Score for Cardiovascular Disease or equivalent). Both trials required LDL-C levels at screening of ≥70 mg/dL (1.8 mmol per liter) in patients with ASCVD, however in the ORION-11 trial, patients with an ASCVD risk equivalent were required to have an LDL-C ≥ 100 mg/dL (2.6 mmol per liter). Patients were stratified in a 1:1 ratio to inclisiran 284 mg or placebo both administered subcutaneously on day 1, day 90, day 270, and day 450. 1561 and 1617 patients underwent randomization in the ORION-10 and ORION-11 trials, respectively. After 510 days of follow-up, inclisiran reduced LDL-C levels by 52.3% (95% CI 48.8–55.7) in the ORION-10 trial and by 49.9% (95% CI 46.6–53.1%) in the ORION-11 trial. There were no significant differences in adverse events though injection site reactions were more frequent with inclisiran and were generally mild. A pooled analysis of ORION-9, -10, and -11 trials demonstrated that the placebo-corrected change in LDL-C with inclisiran at day 510 was −50.7% (95% CI: −52.9% to −48.4%; *p* < 0.0001). Safety was similar between both groups. Treatment-emergent adverse events at the injection site were more frequent with inclisiran than placebo (5.0 vs. 0.7%), however they were mostly mild [96]. The ORION-4 is an outcomes trial currently underway in trial sites across the UK and the USA. Patients with pre-existing ASCVD are being randomized to inclisiran 300 mg vs. placebo both given subcutaneously on day 1, at three months, and then every six months in a 1:1 ratio for planned median duration of approximately five years [95,97].

### 4.2. Target: ANGPTL3

SiRNA therapy targeting ANGPTL3 is also being studied [90,91]. ARO-ANG3 is a GalNAc3-conjugated siRNA which prevents the transcription of ANGPTL3 mRNA in the liver. The phase I/II studies of ARO-ANG3 are underway. Injection of ARO-ANG3 for a duration of 16 weeks at 100, 200, and 300 mg doses reduced levels of ANGPTL3 by 96%, TG by 72%, and LDL-C by 50%. In the FH group, ARO-ANG3 reduced ANGPTL3 levels by 62–92% after 16 weeks in a dose-dependent manner. LDL-C levels were reduced by 23–37%, and TG levels were lowered by 25–43% at doses of 100, 200, and 300 mg. Adverse events were mild. 

### 4.3. Target: Lp (a)

In the APOLLO trial, an siRNA was studied among five centers in the USA, UK, and Australia. This double-blinded placebo-controlled single ascending dose study analyzed adult subjects who were otherwise healthy with an Lp (a) level  ≥ 150 nmol/L to establish Lp (a) levels after 150 days of follow-up [98]. SLN360, an siRNA targeting apolipoprotein (a) synthesis, was subcutaneously administered attached to a sugar, GalNaC (to assist in transport to the liver). After being cleaved, one of the RNA strands removes the messenger RNA thereby preventing translation of the apo (a) protein required for Lp (a) formation [98]. The maximal median reduction in Lp (a) was 20, 89, 185, 268, and 227 nmol/L for those treated with placebo and the four SLN260 doses, respectively. Almost complete eradication of Lp (a) was noted in a dose-dependent manner with Lp (a) reduction of 98% and 96% at the top two doses of SNL360 which persisted at 150 days. LDL-C was also reduced with a 21% and 26% reduction in the two highest doses and apoB was also reduced. These reductions lasted for 150 days.

The OCEAN [a]-DOSE trial tested Olpasiran, an siRNA targeting Lp (a). This multicenter, randomized, double-blind, placebo-controlled trial took place at 34 sites in seven countries [99]. 281 patients were randomized in a 1:1:1:1:1 ratio to varying doses of olpasiran (10 mg every 12 weeks, 75 mg every 12 weeks, 225 mg every 12 weeks, or 225 mg every 24 weeks) or placebo, administered subcutaneously. Hence, 88% of the patients were on statin therapy, 52% on ezetimibe, and 23% were on a PCSK9 inhibitor when the trial began enrolling patients. At 36 weeks, olpasiran reduced the Lp (a) level in a dose-dependent manner, with placebo-adjusted mean percent changes of −70.5% with the 10-mg dose, −97.4% with the 75-mg dose, −101.1% with the 225-mg dose administered every 12 weeks, and −100.5% with the 225-mg dose administered every 24 weeks (*p* < 0.001 for all as compared to baseline). Side effects were equivalent across the trial groups. The placebo-adjusted mean percent change in the LDL-C concentration at 36 weeks ranged from −22.6% to −24.8% at the varying the olpasiran dose levels. A CV outcomes trial focusing on olpasiran is currently underway [100].

### 4.4. Target: ApoC3

ARO-APOC3 is an investigational siRNA directed towards the target ApoC3. In a phase 1 study, the safety and pharmacodynamic effects of ARO-APOC3 were assessed in healthy volunteers and participants with hypertriglyceridemia (with a history of fasting serum triglycerides of at least 300 mg/dL (3.38 mmol/L) at screening or prior diagnosis of familial chylomicronemia syndrome (FCS) [101,102,103]. Multiple-dose, double-blind, randomized, placebo control trials in patients with severe hypertriglyceridemia or FCS are ongoing (see Table 3) [104,105].

## 5. Gene Editing: Clustered Regularly Interspaced Short Palindromic Repeats (CRISPR)

As the name suggests, CRISPR is an immune system that was identified in bacteria involving short segments of DNA in palindromes (i.e., identical sequences forwards and backwards) with segments of unique spacer DNA in between the palindromic segments. CRISPR is a system bacteria use to breakdown viral DNA by saving spacer memory of prior bacteriophage infectious DNA. There are also CRISPR associated genes (cas genes) which make helicases and nucleases which unwind and trim the DNA, respectively. CRISPR cas9 most famously known, was identified in strep pyogenes genes and includes CRISPR RNA and tracer RNA as well. Using a guiding segment of RNA, the cas9 system finds the gene of interest and cuts the DNA at that sequence and breaks the gene. There is also the capability to insert a new gene to allow a repair mechanism. CRISPR/cas9 has several uses in medicine, agriculture, and biotechnology subject to ongoing investigation [106].

### Target: PCSK9

CRISPR has been applied in the realm of lipid management targeting the PCSK9 gene. Sequences within the PCSK9 gene are designated sites where CRISPR technology disrupts splicing resulting in dysfunctional PCSK9 protein destined to be degraded. The CRISPR system uses an adenine-based editor compromised of a nickase Cas9, and a deoxyadenosine deaminase domain. Instead of the typically utilized viral vectors, lipid nano particles serve as the delivery system enabling uptake by the liver where PCSK9 is produced. Proof of concept in vivo analysis of this system in cynomolgus monkeys demonstrated an approximately 90% mean decrease in PCSK9 levels and a 60% mean decrease in LDL-C levels, which were sustained for at least eight months after treatment. There were no serious adverse events and only a mild increase in liver enzymes was noted which resolved after two weeks. Off-target editing occurred at mean of less than 1%. However, the site where this occurred shares very little homology with the human genome, so off-target editing is not anticipated but will need to be verified in long-term human studies. If successful, this approach may allow the possibility of once in a lifetime treatment for dyslipidemia [107,108].

An open-label, phase 1b, single ascending dose study (VT-1001) is underway to assess the safety of a CRISPR based gene editing therapy, VERVE-101, in patients with heterozygous FH, ASCVD, and refractory hypercholesterolemia [109].

## 6. Conclusions

While an estimated 80% of CVD is preventable, CVD continues to be the number one cause of death worldwide [110,111]. The fast-paced advancements in lipid lowering therapies have broadened treatment options to include monoclonal antibodies, ASOs, siRNAs, and single use gene editing therapies under investigation and on the treatment horizon. Targeting the PCSK9 protein, the monoclonal antibodies alirocumab and evolocumab have changed the landscape of LDL-C management for statin intolerant, treatment refractory, and high-risk patients with significantly elevated cholesterol levels, especially in the setting of FH. In a similar vein, siRNA based inclisiran has reimagined the possibilities of maintaining LDL-C levels at target for these high-risk patients in a way that allows for a longer dosing interval at every six months. ASO based mipomersen offers an alternative pathway to treat FH by targeting apoB-100. Moreover, other ASO and siRNA-based therapies remain under active investigation targeting Lp (a), ANGPTL3, and APOC3. Real-world efficacy and CV outcomes trials remain to be performed. Finally, CRISPR based gene editing technology is a paradigm shift towards one time therapy to achieve the lifelong control of lipids. Current efforts are focused on the PCSK9 target. There is a critical need to prevent ASCVD. While lifestyle changes are crucial to reduce risk, modern day therapies to improve lipid control are powerful tools to add on to existing therapies. These therapies have also moved genetics from being a traditionally nonmodifiable ASCVD risk factor towards an imaginable future with empowering opportunity.

## Figures and Tables

**Table 1:** Summary of monoclonal antibodies currently available or under investigation.

Target Protein	FDA Approved Therapies	Therapy Under Investigation	Efficacy	Safety
PCSK9	Evolocumab and Alirocumab	Oral PCSK9 inhibitor MK-0616	FOURIER and FOUIER-OLE trials: Patients on evolocumab in the parent trial and continued its use in the FOURIER-OLE trial had 23% lower risk of CV death as compared to patients who took placebo in the parent trial (HR 0.77, 95% CI 0.60–0.99, *p* = 0.04). ODYSSEY OUTCOMES trial: there was a significant reduction in MACE for patients on alirocumab vs. placebo (9.5% vs. 11.1%, HR 0.85, 95% CI 0.78–0.93, *p* < 0.001.	Mild injection site reactions.
ANGPTL3	Evinacumab		Meta-analysis of 5 RCTs: evinacumab reduced LDL-C significantly compared with placebo [MD −33.12%, 95% CI, −48.63% to −17.60%, *p* < 0.0001], triglycerides (MD −50.95%, 95% CI, −56.55% to −45.36%, *p* < 0.0001), and HDL-C (MD −12.77%, 95% CI, −16.35% to −9.18%, *p* < 0.0001). CV outcomes data unknown	Adverse events did not significantly differ in treatment vs. placebo groups.

**Table 2:** Summary of antisense oligonucleotide therapy currently available or under investigation.

Target Protein	FDA Approved Therapies	Therapy Under Investigation	Efficacy	Safety
PCSK9	None	AZD8233	ETESIAN phase 2b study: LDL-C and PCSK9 levels were reduced in a dose dependent manner at week 12 for patients on study drug vs. placebo. LDL-C decreased −72% (95% CI −78 to −65) for the 50 mg dose and −79% (95% CI −83 to −74) for the 90 mg dose.	No significant adverse events.
			CV outcomes unknown.	No significant adverse events.
ANGPTL3	None	Vupanorsen	TRANSLATE-TIMI trial: Vupanorsen achieved a decrease in non-HDL-C up to 27.7% in the 80 mg every 2 weeks arm (*p* < 0.001). Triglycerides were reduced up to 56.8% (*p* < 0.001) in a dose dependent manner and LDL-C was reduced up to 16% without a dose dependent association.	
Lp (a)	None	Pelacarsen	Phase IIb trial: Significant reductions in direct Lp (a) cholesterol in a dose-dependent manner were observed with pelacarsen compared with pooled placebo, by a mean of 29–67% versus 2% respectively, *p* = 0.001. Pelacarsen was also associated with a modest decrease in laboratory-reported LDL-C.	No significant adverse events.
ApoC3	None	Volanesorsen (approved in EU)	Pooled analysis of four studies showed significant reduction in TG after 3 months of treatment with volanesorsen compared with placebo (MD: −73.9%; 95%CI: −93.5%, −54.2; *p* < 0.001 I2 = 89.05%; *p* < 0.001) with significant decrease in LDL-C and increase in HDL-C as well.	Safety concerns related to thrombocytopenia and bleeding.
apoB-100				
	Mipomersen			
		None		
			During phase I, II and III trials, mipomersen significantly lowered LDL-C, apoB-100 and Lp(a) from baseline. In a phase 3 multicenter blinded randomized placebo-controlled study, mipomersen reduced LDL-C by −36.9% as compared to the placebo group at −4.5% (*p* < 0.001).	Most common side effects include injection site reactions, flu-like symptoms and hepatotoxicity.

**Table 3:** Summary of short interfering RNA currently available or under investigation.

Target Protein	FDA Approved Therapies	Therapy Under Investigation	Efficacy	Safety
PCSK9	Inclisiran		A pooled analysis of ORION-9, 10, and 11 trials demonstrated that the placebo-corrected change in LDL-C with inclisiran at day 510 was −50.7% (95% CI: −52.9% to −48.4%; *p* < 0.0001). CV outcomes trial currently ongoing.	Treatment-emergent adverse events at the injection site were more frequent with inclisiran than placebo (5.0 vs. 0.7%), but were predominantly mild
ANGPTL3		ARO-ANG3	Phase I/II clinical Subcutaneous injection of ARO-ANG3 for 16 weeks at doses of 100, 200, and 300 mg reduced circulating levels of ANGPTL3 by 96%, TG by 72%, and LDL-C by 50%. In the FH group, LDL-C levels were reduced by 23%–37%, and TG levels were reduced by 25%–43% at doses of 100, 200, and 300 mg injected subcutaneously.	Most adverse events were mild.
Lp(a)		Olpasiran	OCEAN [a]-DOSE trial: At 36 weeks, olpasiran reduced the Lp(a) concentration in a dose-dependent manner. The placebo-adjusted mean percent change in the LDL-C concentration at 36 weeks ranged from −22.6% to −24.8% across the olpasiran dose levels. A CV outcomes trial is currently underway.	The overall incidence of adverse events was similar across the trial groups.
APOC3		ARO-APOC3	Multiple dose double-blind randomized placebo control trials in patients with severe hypertriglyceridemia or FCS are ongoing.	No significant adverse events noted.

## Data Availability

Not applicable.
